# Radiological pure-solid appearance clinical stage I lung adenocarcinoma: a comparative study of mucinous and non-mucinous adenocarcinoma based on imaging features and survival outcomes

**DOI:** 10.1186/s13244-026-02353-x

**Published:** 2026-07-23

**Authors:** Wei Li, He Du, Shixing Wu, Jingyi Wang, Nan Song, Ziwei Wan, Fengying Wu, Jingyun Shi

**Affiliations:** 1 Department of Radiology, Shanghai Pulmonary Hospital, Tongji University School of Medicine, Shanghai, China; 2 Department of Oncology, Shanghai Pulmonary Hospital, Tongji University School of Medicine, Shanghai, China; 3 Department of Thoracic Surgery, Shanghai Pulmonary Hospital, Tongji University School of Medicine, Shanghai, China

**Keywords:** Adenocarcinoma, Diagnostic imaging, Epidermal growth factor receptor, Lung neoplasms, Prognosis

## Abstract

**Objectives:**

Our study aims to compare the clinicopathological, genetic, and prognostic differences between pure-solid appearance clinical stage I mucinous and non-mucinous adenocarcinomas.

**Materials and methods:**

Five hundred seventy-nine patients with radiological pure-solid appearance clinical stage I lung adenocarcinoma, were retrospectively evaluated. Clinicopathological, radiological, genetic characteristics, and recurrence patterns were compared between the non-mucinous adenocarcinoma and mucinous adenocarcinoma groups. Recurrence-free survival (RFS) and overall survival (OS) were assessed by Kaplan–Meier curves and compared between groups using log-rank tests. Cox proportional hazard models were used to analyze prognostic factors.

**Results:**

Of 579 patients (mean age, 59.9 years ± 9.7 [SD]; 298 women), 442 had non-mucinous adenocarcinoma, and 137 had mucinous adenocarcinoma. The non-mucinous adenocarcinoma group, in comparison with mucinous adenocarcinoma, showed higher CEA levels (*p* = 0.003), higher CT values (36.88 vs 20.89 HU, *p* < 0.001), a higher incidence of emphysema (25.1% vs 3.6%, *p* < 0.001), a lower clinical T stage (51.8% vs 42.3%, *p* < 0.001), and the absence of air bronchogram (61.3% vs 27%, *p* < 0.001). Recurrences occurred more frequently in the non-mucinous adenocarcinoma group (38.5%, 170/442) compared to the mucinous adenocarcinoma group (13.1%, 18/137; *p* < 0.001). Brain metastasis was significantly more common in the non-mucinous adenocarcinoma group (27.1% vs 0%; *p* = 0.024). Compared with the non-mucinous adenocarcinoma group, the mucinous adenocarcinoma group had better 5-year RFS (86.9% vs 62.7%; *p* < 0.001) and 5-year OS (97.1% vs 83.2%, *p* < 0.001).

**Conclusion:**

Imaging features, survival outcomes, and recurrence patterns differed significantly between mucinous and non-mucinous lung adenocarcinomas.

**Key Points:**

- ***Question*** For radiological pure-solid appearance clinical stage I lung adenocarcinoma, there remains controversy regarding the clinicopathological characteristics and prognosis of mucinous versus non-mucinous subtypes.
- ***Findings*** There were significant differences in clinicopathological features, imaging findings, and recurrence patterns between mucinous and non-mucinous adenocarcinomas. Compared with the non-mucinous adenocarcinoma, the mucinous group had better 5-year RFS and 5-year OS.
- ***Critical relevance statement*** Lung mucinous adenocarcinoma, Lung mucinous adenocarcinoma, due to its unique imaging and clinicopathological features, was a special independent subtype of adenocarcinoma. For radiological pure-solid appearance lung adenocarcinoma, mucinous adenocarcinoma had a better survival prognosis than non-mucinous adenocarcinoma.

**Graphical Abstract:**

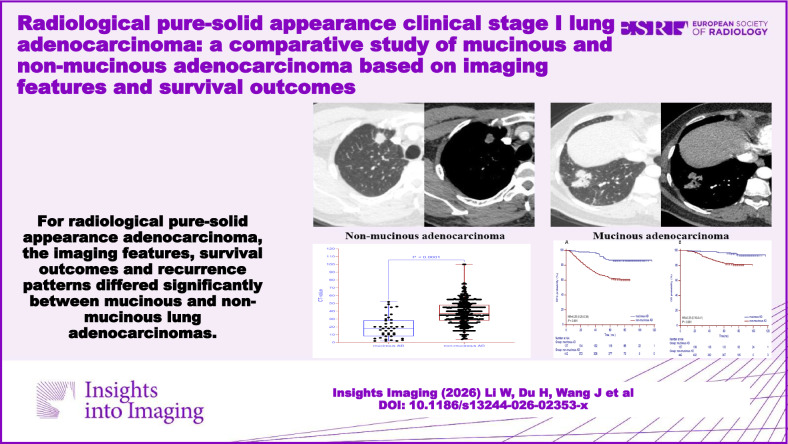

## Introduction

Ground-glass opacity (GGO) is defined as focal nodular lesions of increased attenuation without internal solid components, with the preservation of bronchial and vascular margins on high-resolution computed tomography (HRCT) scans [1]. Previous studies have demonstrated that the GGO and consolidation on HRCT findings are mostly correlated with the lepidic growth and invasive component on pathology, respectively [2, 3]. Furthermore, a large number of studies have confirmed that sub-solid nodules containing GGO components have a better prognosis compared to pure-solid lung adenocarcinoma [4–7]. Our previous study also confirmed that even if it contains a minor amount of GGO components, the survival outcome is significantly superior to that of pure-solid lung adenocarcinoma [8]. Patients with pure-solid or sub-solid tumors on imaging show significant differences in the clinicopathologic and oncologic outcomes, which may reflect the distinct carcinogenesis of these two types of lesions [9]. Therefore, some researchers have proposed that sub-solid nodules containing GGO components represent a special clinical subtype of lung adenocarcinoma [10]. Compared with the sub-solid nodule, the pure-solid appearance adenocarcinoma may have a different growth pattern and origin. Moreover, it grows faster and has a poorer prognosis, it would be a clinical dilemma of how these lesions should be recognized. Radiologically, pure-solid appearance lung adenocarcinoma is sometimes relatively difficult to diagnose precisely in imaging because it does not contain GGO components. However, we can perceive it through non-intuitive parameters on HRCT, such as the texture and density of the tumor.

In the 2011 lung adenocarcinoma classification system proposed by the International Association for the Study of Lung Cancer (IASLC), American Thoracic Society (ATS), and European Respiratory Society (ERS), primary lung mucinous adenocarcinoma (LMA) was defined as a distinct variant of lung adenocarcinoma [2]. Invasive mucinous adenocarcinoma, previously termed mucinous bronchioloalveolar carcinoma (BAC), is histologically distinctive among primary lung cancers, characterized by columnar or goblet cell morphology, basal nuclei, and a cytoplasmic accumulation of mucin [11]. The differentiation between LMA and non-mucinous adenocarcinoma primarily stems from notable variations in clinical presentation, imaging characteristics, pathology, and genetic features [12–15]. Pneumonic type LMA has relatively typical imaging features, often manifesting as consolidation with multiple lobe involvements and a tendency to spread through the airway lumen [16]. When the imaging shows a pure-solid nodule, there are no intuitive parameters to distinguish mucinous adenocarcinoma from non-mucinous adenocarcinoma. However, we can sense this difference; for instance, mucinous adenocarcinoma presenting as pure-solid nodules is relatively softer due to the presence of mucin. To our knowledge, there has been no previous research to confirm how to distinguish these two pathological subtypes of lung adenocarcinoma through imaging features. In addition, a comparison of survival outcomes and recurrence patterns between invasive mucinous and non-mucinous adenocarcinomas, incorporating HRCT features and pathological characteristics, remains unexplored.

Thus, this study aims to compare the clinicopathological, genetic, and prognostic differences between pure-solid appearance clinical stage I mucinous and non-mucinous adenocarcinomas and to examine the potential underlying factors from a multifaceted perspective.

## Materials and methods

### Study sample

This retrospective study was approved by the Institutional Review Board of Shanghai Pulmonary Hospital (IRB No. K25-871), which waived the requirement for informed consent from patients. Between January 2015 and February 2017, a radiologist (W.L., with 13 years of experience in chest radiological diagnosis) conducted a search using terms such as “pure-solid nodule or mass” and “possibly malignant” in the hospital’s radiological information system, identifying a total of 1560 patients for potential inclusion. After applying the following exclusion criteria, 981 patients were excluded: (a) tumors exceeding 4 cm in size; (b) mediastinal or hilar lymph nodes with a short-axis diameter greater than 1 cm; (c) pathology confirming non-adenocarcinoma; (d) recurrent or metastatic tumors; (e) a history of previous malignancy; (f) incomplete clinical or radiological data; (g) preoperative CT scans without contrast enhancement, as differing CT parameters could introduce interpretation bias, particularly regarding CT attenuation; and (h) patients lost to follow-up or with less than 5 years of follow-up. Ultimately, 579 patients with radiologically confirmed pure-solid adenocarcinomas were included in this study.

The following clinical features were recorded for all patients: age at diagnosis, gender, smoking history, carcinoembryonic antigen (CEA) levels, and surgical procedures. Clinical T stage for each tumor was assigned according to the 8th edition of the tumor–node–metastasis (TNM) classification for lung cancer.

### Chest HRCT protocol and radiological evaluation

Chest HRCT was conducted using the following scanners: Somatom Definition, Sensation-16 (Siemens Medical Solutions), and Brilliance 64 (Philips Medical Systems), with settings of 120 kVp, 100–200 mAs, pitch ranging from 0.875 to 1.5, and collimation of 0.625–1.0 mm. Images were reconstructed using a medium-sharp reconstruction algorithm, with a slice thickness of 0.625–1.0 mm. Intravenous administration of 100 mL of nonionic contrast material (Lopamiro) was performed at a rate of 4.0–5.0 mL/s. A region of interest was placed on the aorta, and craniocaudal scanning commenced 5 s after the attenuation reached 120 Hounsfield units (HU), spanning from the lung apex to the lung base during a single breath-hold.

All HRCT scans were independently reviewed by two chest radiologists (W.L. and J.S., with 13 and 31 years of experience in chest radiological diagnosis), who were blinded to the study’s purpose. Evaluations were based on lung and mediastinal window settings, with a lung window level of −450 HU and width of 1500 HU, and a mediastinal window level of 40 HU and width of 400 HU. The following CT parameters were recorded: emphysema, tumor distribution and location, CT attenuation, and presence of air bronchograms. Clinical T categories were determined based on the maximal tumor size in any of the three planes, according to the eighth edition of the TNM classification.

### Pathological evaluation

All tumors were fully sampled and submitted for histopathological evaluation following surgical resection. The predominant histologic subtypes were identified according to the IASLC/ATS/ERS classification of lung adenocarcinomas. Pathological features, including nodal involvement, visceral pleural invasion (VPI), and lymphatic/vascular invasion (LVI), were also assessed. Additionally, genetic data regarding EGFR mutations, ALK rearrangements, KRAS, BRAF, and ROS1 status were extracted from the pathological reports.

### Follow-up for survival analysis and analysis of recurrence patterns

Following the resection of lung tumors, patients were scheduled for follow-up examinations at 3 months post-surgery, then at 6-month intervals during the first 2 years, and annually thereafter until 5 years. Survival and disease progression were assessed through medical records or telephone interviews. Recurrence was diagnosed based on physical examination and diagnostic imaging, with histological confirmation when clinically feasible. All follow-up chest CT scans and other imaging studies were retrospectively reviewed by a radiologist (W.L., with 13 years of experience in chest radiological diagnosis) and a thoracic oncologist (H.D., with 12 years of experience in the treatment of thoracic oncology). Recurrence-free survival (RFS) and overall survival (OS) were defined as the time from surgical resection to the first recurrence of lung cancer (local, regional, or distant metastasis) or death from any cause, and from surgery to death from any cause, respectively. For patients who were alive or had no recurrence at the time of the last visit/contact, RFS and OS were censored at the date of the last disease assessment.

Local recurrence was defined as tumor regrowth near the staple line or bronchial stump. Regional recurrence referred to recurrence within the ipsilateral lung or lymph node stations 1–14, as well as the bilateral supraclavicular fossa. Distant metastasis was defined as metastasis to the pleura, pericardium, contralateral lung, other lymph node stations, or extrathoracic sites.

### Statistical analysis

Clinicopathological characteristics and HRCT findings were presented as mean ± standard deviation (SD) or frequency (percentage), as appropriate. Differences between groups were assessed using one-way analysis of variance (ANOVA) for continuous variables, and the Chi-squared test or Fisher’s exact test for categorical variables.

Kaplan–Meier curves were constructed to depict the cumulative probability of experiencing events (RFS and OS) over time, with group differences analyzed using the log-rank test. Hazard ratios (HRs) with 95% confidence intervals (CIs) for prognosis were estimated using a stepwise Cox proportional-hazards model. The proportional hazards assumption was tested using Schoenfeld’s global test. Variables were included in the multivariable Cox regression model based on their univariate associations with outcomes and clinical relevance.

All statistical analyses were conducted using SPSS version 20.0 (IBM) and MedCalc version 11.2.1.0 (MedCalc Software). A two-sided *p* value of < 0.05 was considered statistically significant.

## Results

### Patient demographic and clinical characteristics

**Fig. 1:**
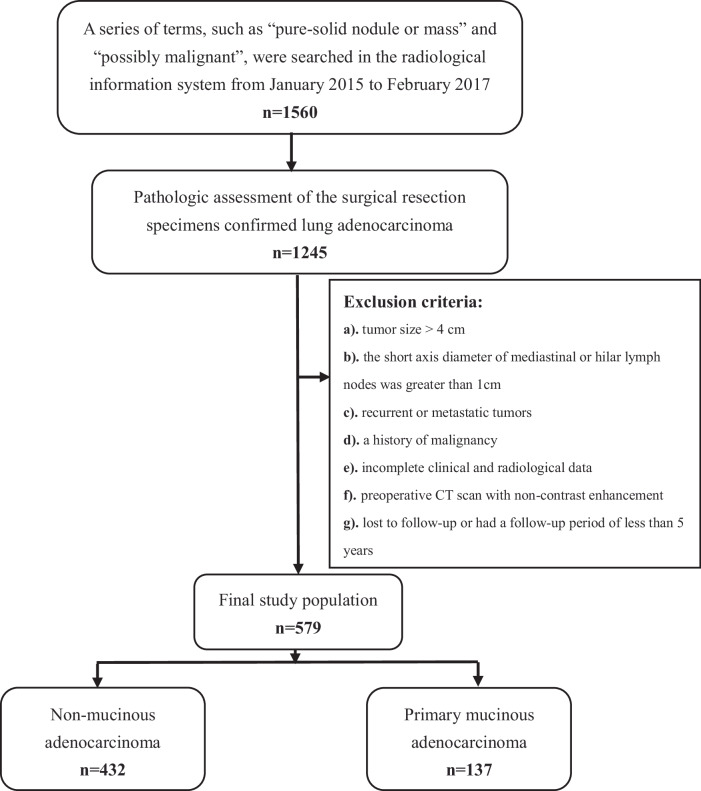
The flow diagram shows patient selection and exclusion criteria

Among the 1560 patients identified with radiologically pure-solid lung cancers, 579 patients (mean age: 59.9 ± 9.7 years; 298 women) were included in the final study cohort (Fig. 1). The mean tumor size was 2.1 ± 0.7 cm. Most patients were non-smokers (79.3%, 459/579). Of the total sample, 442 patients were classified into the non-mucinous adenocarcinoma group, and 137 into the mucinous adenocarcinoma group. Tumor location analysis revealed that 63.5% (87/137) of mucinous adenocarcinomas were in the lower lobe, while 50.3% (222/442) of non-mucinous adenocarcinomas were in the upper lobe. Additionally, most tumors (97.4%, 564/579) were peripherally distributed. According to the 8th edition of the TNM classification, the clinical T stages were as follows: 45 patients had cT1a, 242 had cT1b, 223 had cT1c, and 69 had cT2a disease. Lobectomy was performed in 87.2% (505/579) of cases, and the majority (95.7%, 554/579) underwent systematic lymph node dissection. All patients had negative resection margins. Furthermore, 49.4% (286/579) of patients received adjuvant therapy (Table 1).

**Table 1:** Demographic and clinical characteristics of the total study sample (*n* = 579)

Variable	Value
Age (y)^*^	59.9 ± 9.7
Gender	
Male	281 (48.5)
Female	298 (51.5)
Smoking history	
Never smoker	459 (79.3)
Ex-smoker or current smoker	120 (20.7)
CEA^*^	2.12 (1.25–4.64)
Tumor distribution	
Central	15 (2.6)
Peripheral	564 (97.4)
Tumor location	
Left upper lobe	117 (20.2)
Right upper lobe	142 (24.5)
Right middle lobe	63 (10.9)
Right lower lobe	130 (22.5)
Left lower lobe	127 (21.9)
Diameter (cm)^*^	2.1 ± 0.7
CT value^*^	33.1 ± 17.2
Emphysema	
Yes	116 (20.0)
No	463 (80.0)
Air bronchogram	
Yes	271 (46.8)
No	308 (53.2)
Clinical T stage	
T1a (≤ 1 cm)	45 (7.8)
T1b (1–2 cm)	242 (41.8)
T1c (2–3 cm)	223 (38.5)
T2a (3–4 cm)	69 (11.9)
Genic alteration	
EGFR	237 (48.8)
KRAS	66 (13.6)
ALK	48 (9.9)
Wild type	129 (26.5)
Other	6 (1.2)
Visceral pleural involvement (+)	95 (16.4)
Lymphatic/vascular involvement (+)	18 (3.1)
Pathological nodal involvement (+)	107 (18.5)
Operative procedure	
Lobectomy	505 (87.2)
Non-lobectomy	74 (12.8)
Overall Recurrence	188 (32.5)
Cancer-related death	86 (14.9)
Follow-up interval (month)^*^	73 (67-83)
Adjuvant therapy	
Yes	286 (49.4)
No	293 (50.6)

*CEA* carcinoembryonic antigen, *EGFR* epidermal growth factor receptor, *ALK* anaplastic lymphoma kinase, *KRAS* Kirsten rat sarcoma viral oncogene

^*^ Data are medians, with interquartile ranges in parentheses

### Comparison of clinicopathological, radiological, and genetic characteristics according to histologic subtypes

**Fig. 2:**
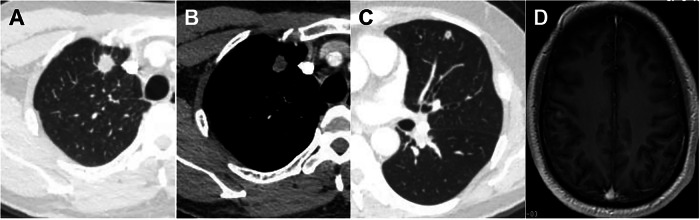
Images in a 50-year-old man with distant metastasis after surgical resection of acinar-predominant adenocarcinoma in clinical stage I. **A**, **B** Axial contrast-enhanced CT image with a lung window setting shows a 15 mm spiculated pure-solid nodule in the right upper lobe. The preoperative clinical staging of the patient was T1bN0M0, with an overall stage of IA2. The lesion was pathologically confirmed as acinar-predominant adenocarcinoma without lymph node metastasis and lymphatic and vascular involvement (LVI), but with VPI. Therefore, the postoperative pathological staging of the patient was T2aN0M0, with an overall stage of IB. Genetic evaluation indicated that the lesion carried an *EGFR* mutation. **C**, **D** Two years after the surgery, postoperative examination revealed distant metastases, including multiple pulmonary pure-solid nodules, rib metastases, and brain metastasis. Cancer-specific death had occurred after 68 months of follow-up

**Fig. 3:**
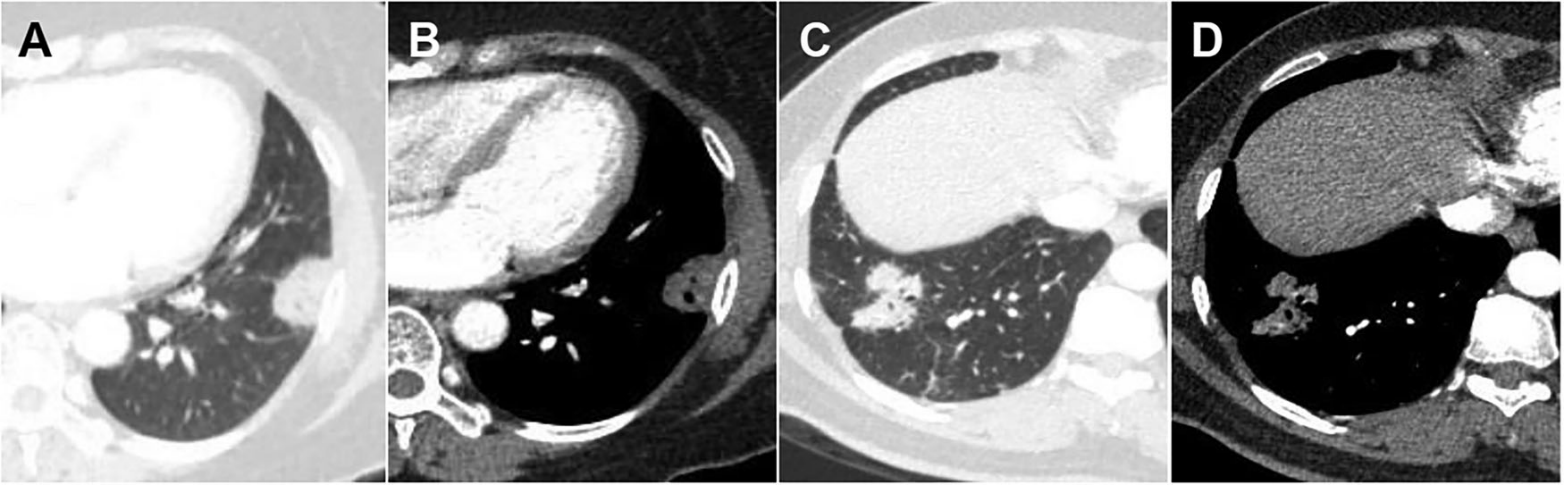
**A**, **B** Images in a 59-year-old woman with primary LMA in clinical stage I. Axial contrast-enhanced CT image shows a 29-mm well-defined lobulated pure-solid nodule with an air bronchogram sign in the left lower lobe. The CT attenuation of the lesion was 20 HU. **C**, **D** Images in a 55-year-old woman with primary LMA in clinical stage I. Axial contrast-enhanced CT image shows a 39-mm well-defined lobulated pure-solid nodule with an air bronchogram sign in the right lower lobe. The CT attenuation of the lesion was 22Hu. Genetic analysis indicated that these two patients carry KRAS mutation, and there was no recurrence or death within 5 years after surgery

**Fig. 4:**
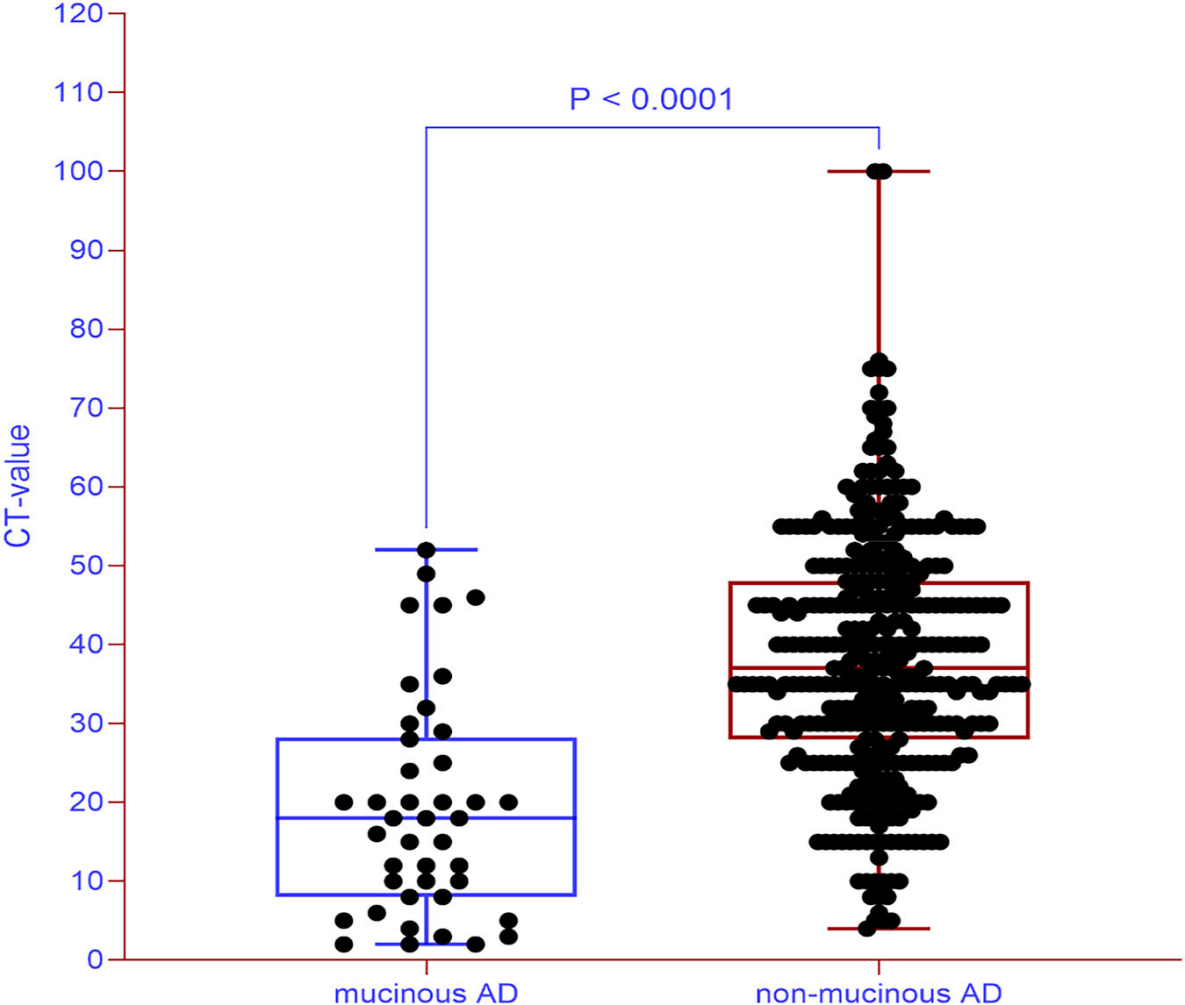
Box and whisker plots showed the distribution of CT values according to pathological subtypes. The median, upper whisker, and lower whisker CT values of the non-mucinous adenocarcinoma group were significantly higher than those of the mucinous adenocarcinoma group. AD, adenocarcinoma

Clinicopathological, radiological, and genetic characteristics by histologic subtype are summarized in Table 2, with examples of pure-solid appearance lung adenocarcinomas shown in Figs. 2 and 3. Compared to the mucinous adenocarcinoma group, the non-mucinous adenocarcinoma group exhibited a significantly higher proportion of males (52.7% vs 35.0%, *p* < 0.001) and a greater incidence of smoking history (22.9% vs 13.9%, *p* = 0.023), with no significant age difference (*p* > 0.05). The non-mucinous adenocarcinoma group also had higher CEA levels (*p* = 0.003). The results of interobserver agreement for various CT characteristics of eligible patients were shown in Table S1. All features showed high agreement, with intraclass correlation coefficient (ICC) or kappa coefficients greater than 0.90. Regarding imaging characteristics, the non-mucinous adenocarcinoma group was associated with higher CT attenuation values (36.9 vs 20.9 HU, *p* < 0.001), a higher incidence of emphysema (25.1% vs 3.6%, *p* < 0.001), a lower clinical T stage (51.8% vs 42.3%, *p* < 0.001), and the absence of air bronchogram (61.3% vs 27.0%, *p* < 0.001). The box-and-whisker plots displayed the distribution of CT attenuation across pathological subtypes (Fig. 4), showing significantly higher median, upper whisker, and lower whisker CT values in the non-mucinous adenocarcinoma group (*p* < 0.001). No significant differences were found in the maximum tumor diameter or lesion distribution between the two groups (both *p* > 0.05). Pathological nodal involvement and VPI were more frequently observed in the non-mucinous adenocarcinoma group (21.5% vs 8.8%, *p* = 0.001; 21.0% vs 2.9%, *p* < 0.001), while no significant difference in LVI was observed (*p* = 0.12). Pathological stage upgrading after surgery was also more common in the non-mucinous adenocarcinoma group (*p* < 0.001). Genetic analysis results were available for 486 of 579 patients. EGFR mutations were more prevalent in the non-mucinous adenocarcinoma group, while KRAS mutations were more frequent in the mucinous adenocarcinoma group (*p* < 0.001).

**Table 2:** Comparison of clinicopathological, radiological, and genetic characteristics according to histologic subtypes

Variables	Non-mucinous AD (*n* = 442)	Mucinous AD (*n* = 137)	*p* value
Gender			0.000^b^
Male	233 (52.7)	48 (35.0)	
Female	209 (47.3)	89 (65.0)	
Age (y)	60.19 ± 9.53	58.87 ± 10.05	0.162^a^
Smoking			0.023^b^
Never	341 (77.1)	118 (86.1)	
Past or current	101 (22.9)	19 (13.9)	
CEA			0.003^b^
≤ 2.24	216 (48.9)	87 (63.5)	
>2.24	226 (51.1)	50 (36.5)	
Emphysema			0.000^b^
No	331 (74.9)	132 (96.4)	
Yes	111 (25.1)	5 (3.6)	
Distribution			0.98^b^
Central	12 (2.7)	3 (2.2)	
Peripheral	430 (97.3)	134 (97.8)	
Location			0.000^b^
Right upper lobe	121 (27.4)	21 (15.3)	
Right middle lobe	50 (11.3)	13 (9.5)	
Right lower lobe	89 (20.1)	41 (29.9)	
Left upper lobe	101 (22.9)	16 (11.7)	
Left lower lobe	81 (18.3)	46 (33.6)	
Diameter (cm)	2.05 ± 0.64	2.21 ± 0.98	0.186^a^
CT value (HU)	36.88 ± 15.7	20.89 ± 15.9	0.000^a^
Air bronchogram	171 (38.7)	100 (73.0)	0.000^b^
Clinical T stage			0.000^b^
T1a (≤ 1 cm)	24 (5.4)	21 (15.3)	
T1b (1–2 cm)	205 (46.4)	37 (27.0)	
T1c (2–3 cm)	182 (41.2)	41 (30.0)	
T2a (3–4 cm)	31 (7.0)	38 (27.7)	
Gene mutation	303 (74.4)	54 (68.3)	0.26^b^
Mutation site			0.000^b^
EGFR	233 (57.2)	4 (5.1)	
KRAS	29 (7.1)	37 (34.2)	
ALK	35 (8.6)	13 (16.5)	
Wild type	104 (25.5)	25 (31.6)	
Other	6 (1.5)	0 (0)	
Visceral pleural involvement	93 (21.0)	4 (2.9)	0.000^b^
Lymphatic/vascular involvement	17 (3.8)	1 (0.73)	0.12^b^
Pathological nodal involvement	95 (21.5)	12 (8.8)	0.001^b^
Pathological stage			0.000 ^b^
IA1	23 (5.2)	21 (15.3)	
IA2	131 (29.6)	35 (25.5)	
IA3	110 (24.9)	33 (24.1)	
IB	79 (17.9)	35 (25.5)	
IIA	5 (1.1)	1 (0.7)	
IIB	32 (7.2)	3 (2.2)	
IIIA	62 (14.0)	9 (6.6)	
Postoperative treatment	202 (45.7)	84 (61.3)	0.001^b^
Overall recurrence	170 (38.5)	18 (13.1)	0.000^b^
Cancer-related death	79 (17.9)	7 (5.1)	0.000^b^

Unless otherwise specified, data are numbers of patients, with percentages in parentheses. Percentages may not add up to 100 because of rounding.

*AD* adenocarcinoma, *CEA* carcinoembryonic antigen, *HU* Hounsfield unit, *EGFR* epidermal growth factor receptor, *KRAS* Kirsten rat sarcoma viral oncogene, *ALK* anaplastic lymphoma kinase

*a* oneway analysis of variance (Wilcoxon W)

*b* chi-square test

### Imaging predictors of non-mucinous adenocarcinoma and mucinous adenocarcinoma in c-stage I pure-solid appearance lung adenocarcinoma

**Fig. 5:**
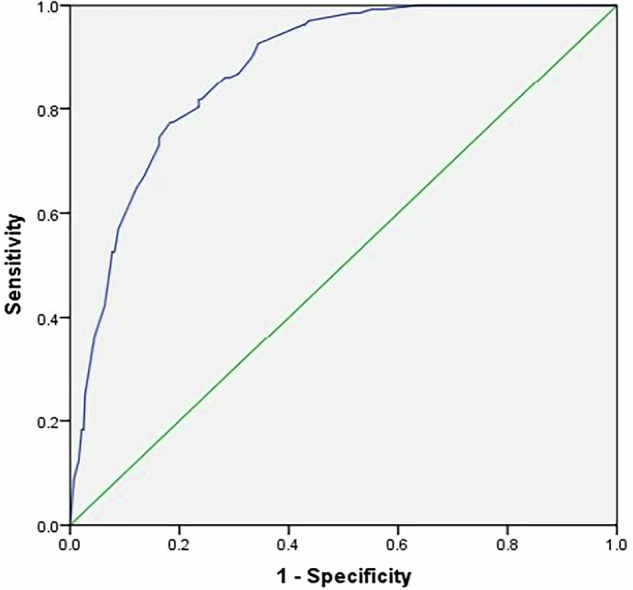
Receiver operating characteristic curves show that the mean area under the curve (AUC) was 0.876 in the model that used the imaging features for predicting mucinous adenocarcinoma

Among patients with either non-mucinous adenocarcinoma or mucinous adenocarcinoma, when holding the other covariates fixed, the odds of mucinous adenocarcinoma group were significantly greater for the patients with air bronchogram in the primary tumor (odds ratio [OR], 3.7; 95% confidence interval [CI]: 2.2–6.2, lower lobe (OR, 2.4; 95% CI: 1.5–3.8), than for those without these features. In contrast, the odds of the mucinous adenocarcinoma group were significantly lower for the patients with high CT attenuation (CT ≥ 34 Hu) (OR, 0.1; 95% CI: 0.07–0.2), and the presence of emphysema (OR, 0.1; 95% CI: 0.04–0.3), than for those without these features (Table 3). In logistic regression, the CT value was dichotomized at the median of 34 HU. The receiver operating characteristic curve for the multivariable logistic regression model is shown in Fig. 5.

**Table 3:** Multivariable logistic regression model for non-mucinous adenocarcinoma versus mucinous adenocarcinoma

Variables	OR (95% CI)	*p* value
Air bronchogram [absence (ref.)]	3.7 (2.2–6.2)	0.000
Emphysema [negative (ref.)]	0.1 (0.04–0.3)	0.000
CT value [< 34 Hu (ref.)]	0.1 (0.07–1.0)	0.000
Location, [non-lower lobe (ref.)]	2.3 (1.5–3.8)	0.000
c-T stage, [c-T1a (ref.)]		0.000
c-T1b	0.2 (0.1–0.5)	0.000
c-T1c	0.3 (0.1–0.6)	0.003
c-T2a	1.7 (0.6–4.9)	0.285

*OR* odds ratio, *CI* confidence interval, *CT* computed tomography, *HU* Hounsfield unit

### Comparison of recurrence patterns according to histologic subtypes

Of the 579 patients, 188 experienced tumor recurrence. Recurrence was diagnosed in 35 patients (18.6%) through pathological confirmation and in 153 patients (81.4%) through radiological evaluation. Recurrences occurred more frequently in the non-mucinous adenocarcinoma group (38.5%, 170/442) compared to the mucinous adenocarcinoma group (13.1%, 18/137; *p* < 0.001) (Table 4). The most common recurrence type was distant metastasis, occurring in 94.7% (161/170) of patients with non-mucinous adenocarcinoma and 94.4% (17/18) of patients with mucinous adenocarcinoma. Brain metastasis was significantly more common in the non-mucinous adenocarcinoma group (27.1% vs 0%; *p* = 0.02). While the incidence of pleural metastasis was higher in the non-mucinous group, no statistically significant difference was observed. In the mucinous adenocarcinoma group, recurrences were primarily located in the contralateral lung (72.2%, 13/18), with no brain metastases (Table 4). Additionally, Table S2 shows a higher proportion of patients with brain metastases and pleural invasion carrying EGFR mutations (69.6%, 32/46 and 65.9%, 29/44, respectively).

**Table 4:** Comparison of recurrence patterns according to histologic subtypes

Recurrence pattern	All patients (*n* = 188)	Non-mucinous AD (*n* = 170)	Mucinous AD (*n* = 18)	*p* value
Overall recurrence	188 (32.4)	170 (38.5)	18 (13.1)	0.000
Local recurrence	10 (5.3)	9 (5.3)	1 (5.6)	0.613
Hila or mediastinum	7 (3.7)	6 (3.5)	1 (5.6)	0.824
Supraclavicular fossae	2 (1.1)	2 (1.2)	0 (0)	0.456
Ipsilateral lung	3 (1.6)	3 (1.8)	0 (0)	0.674
Distant metastasis	178 (94.7)	161 (94.7)	17 (94.4)	0.613
Brain	46 (24.5)	46 (27.1)	0 (0)	0.024
Bone	29 (15.4)	24 (14.1)	5 (27.8)	0.127
Contralateral lung	57 (30.3)	44 (25.9)	13 (72.2)	0.000
Other lymph nodes	8 (4.3)	8 (4.7)	0 (0)	0.744
Chest wall or pleura	44 (23.4)	42 (24.7)	2 (11.1)	0.316
Other	10 (5.3)	8 (4.7)	2 (11.1)	0.549

Data are numbers of patients with recurrence, with percentages in parentheses

*AD* adenocarcinoma

If two or more patterns of recurrence appeared at the same time on the date of recurrence, the higher grade of recurrence was selected (distant, then local). If there were two or more initial recurrence sites within each recurrence pattern, they were all recorded

### Survival analysis for RFS

**Fig. 6:**
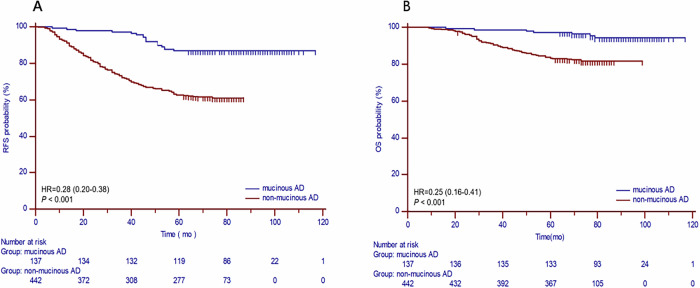
Kaplan–Meier curves according to the pathological subtypes. **A** Five-year RFS and (**B**) OS curves of the mucinous adenocarcinoma group and the non-mucinous adenocarcinoma group. *p* values were obtained by using the log-rank test. AD, adenocarcinoma

The median follow-up time to recurrence was 71 months (range: 1–117 months). Kaplan–Meier survival curves for the non-mucinous and mucinous adenocarcinoma groups are shown in Fig. 6. The 5-year RFS differed significantly between the groups: the mucinous adenocarcinoma group had a better 5-year RFS (86.9%) compared to the non-mucinous adenocarcinoma group (62.7%) (hazard ratio [HR], 0.3; 95% CI: 0.2–0.4; *p* < 0.001).

Univariable analysis indicated that time to recurrence was associated with CT attenuation value, CEA level, tumor distribution, presence of air bronchogram, pathological nodal involvement, LVI, postoperative treatment, and pathological subtype (*p* < 0.05 for all; Table S3). Multivariable Cox regression analysis revealed that the presence of an air bronchogram (HR, 0.5 [95% CI: 0.3–0.6]; *p* < 0.001) and peripheral tumor distribution (HR, 0.5 [95% CI: 0.3–0.9]; *p* = 0.028) were independent predictors of favorable RFS. Conversely, pathological nodal involvement and non-mucinous adenocarcinoma subtype were independent predictors of poor RFS prognosis (HR, 2.6 [95% CI: 1.9–3.6]; *p* < 0.001 and HR, 2.1 [95% CI: 1.2–3.8]; *p* = 0.009; Table 5).

**Table 5:** Multivariable Cox proportional hazard regression analysis of factors affecting RFS and OS

Variables	HR (95% CI)	*p* value
RFS		
Air bronchogram, [absence (ref.)]	0.5 (0.3–0.6)	0.000
Pathological nodal involvement, [negative (ref.)]	2.6 (1.9–3.6)	0.000
Distribution, [central (ref.)]	0.5 (0.3–0.9)	0.028
Postoperative treatment, [no (ref.)]	1.6 (1.1–2.2)	0.008
Histology, [mucinous (ref.)]	2.1 (1.2–3.8)	0.009
OS		
CEA, >2.24 [≤ 2.24 (ref.)]	1.7 (1.1–2.7)	0.018
Pathological nodal involvement, [negative (ref.)]	2.7 (1.7–4.2)	0.000
Air bronchogram, [absence (ref.)]	0.4 (0.2–0.6)	0.000

*HR* hazard ratio, *CI* confidence interval, *CEA* carcinoembryonic antigen

### Survival analysis for OS

The median follow-up time for OS was 73 months (range: 9–117 months), and 86 out of 579 patients (14.9%) died during the follow-up period. The 5-year OS rates were 97.1% in the mucinous adenocarcinoma group and 83.2% in the non-mucinous adenocarcinoma group. Kaplan–Meier curves demonstrated significantly higher 5-year OS in the mucinous adenocarcinoma group compared to the non-mucinous adenocarcinoma group (97.1% vs 83.2%; HR, 0.3 [95% CI: 0.2–0.4]; *p* < 0.001) (Fig. 6).

Univariable analysis identified several factors positively associated with poorer OS: higher CEA levels (*p* = 0.001), non-mucinous adenocarcinoma subtype (*p* < 0.001), pathological nodal involvement (*p* < 0.001), and LVI (*p* = 0.006). Additionally, the presence of an air bronchogram was negatively associated with poorer OS (*p* < 0.001; Table S2). In the final multivariable Cox regression analysis, the presence of an air bronchogram was independently associated with a reduced risk of death (HR, 0.4 [95% CI: 0.2–0.6]; *p* < 0.001). Pathological nodal involvement and higher CEA levels were independent predictors of worse OS (HR, 2.7 [95% CI: 1.7–4.2]; *p* < 0.001 and HR, 1.7 [95% CI: 1.1–2.7]; *p* = 0.018; Table 5).

## Discussion

Pulmonary mucinous adenocarcinoma, due to its distinctive pathological and genetic characteristics, is regarded as a unique variant type of lung adenocarcinoma. It is precisely this difference that leads to significant differences in imaging features, survival outcomes, and recurrence patterns. To our knowledge, the present study is the largest to date to systematically assess mucinous and non-mucinous pure-solid appearance lung adenocarcinoma through imaging parameters, which is a particularly challenging clinical issue. Our study showed that distinct imaging features can help distinguish between the 2 types. Specifically, mucinous adenocarcinomas demonstrated significantly better survival outcomes compared to non-mucinous adenocarcinomas, which were associated with a lower risk of recurrence. Additionally, recurrence patterns varied between the histological subtypes: patients with mucinous adenocarcinomas had a higher incidence of contralateral lung metastasis (72.2%; 13/18) without brain metastasis, while patients with non-mucinous adenocarcinomas were more likely to experience brain metastasis (27.1%; 46/170) and pleural invasion (24.7%; 42/170).

The prognosis comparison between mucinous adenocarcinoma and non-mucinous adenocarcinoma remains controversial among various studies [17–24]. Our study, leveraging a large sample, offers a fresh perspective on the differences between these two groups—an approach not explored in previous research. Consistent with several earlier studies, our findings revealed that the survival outcomes of patients with non-mucinous adenocarcinoma were significantly worse than those with mucinous adenocarcinoma (both *p* < 0.001). The disparity in survival outcomes can be explained through radiological and pathological characteristics. First, the CT attenuation value of mucinous adenocarcinoma was notably lower than that of non-mucinous adenocarcinoma, which could be attributed to the large amounts of mucus present, reducing the CT attenuation. The mucus content likely also contributed to a smaller pathological invasion size [25], and it may limit the metastatic potential of tumor cells to distant sites. Second, air bronchogram was found to be a consistently favorable prognostic factor, with significant associations with both time to recurrence (HR, 0.5; *p* < 0.001) and OS (HR, 0.4; *p* < 0.001), in alignment with previous reports [26, 27]. In our cohort, the incidence of air bronchograms was significantly higher in the mucinous adenocarcinoma group compared to the non-mucinous group (73.0% vs 38.7%, *p* < 0.001). In terms of pathological features, previous studies on the pathological behavior of tumors have established that VPI and pathological lymph node metastasis are negative prognostic indicators in patients with non-small cell lung cancer [10, 28]^.^ Our data corroborate these findings, revealing that the prevalence of VPI, pathological lymph node metastasis, and pathological stage upgrading was significantly higher in the non-mucinous adenocarcinoma group compared to the mucinous adenocarcinoma group. These results suggest that mucinous adenocarcinoma exhibits markedly lower malignant potential than its non-mucinous counterpart.

In this study, recurrence patterns exhibited notable differences between the mucinous and non-mucinous adenocarcinoma groups. In the mucinous adenocarcinoma cohort, recurrences were predominantly observed in the contralateral lung (72.2%, 13/18), with no recorded cases of brain metastasis. In contrast, brain metastasis was significantly more prevalent in the non-mucinous adenocarcinoma group, potentially reflecting underlying molecular genetic disparities. Specifically, EGFR mutations were more frequently observed in the non-mucinous group, whereas KRAS mutations were predominant in the mucinous adenocarcinoma group (*p* < 0.001). Previous research has demonstrated that EGFR is integral to both physiological and pathological angiogenesis, facilitating the formation of angiogenic signals in endothelial and tumor cells, thereby promoting vascular growth through a complex biological cascade [29, 30]. EGFR-induced angiogenesis may enhance the blood supply in EGFR-mutant lung adenocarcinomas, rendering them more susceptible to recurrence and metastasis. In contrast, the pathogenic mechanism of KRAS mutation is different from that of EGFR. Interestingly, our findings also revealed a higher incidence of *EGFR* mutations in patients with brain metastasis, aligning with previous studies [31, 32]. Additionally, postoperative pleural metastasis occurred more frequently in the non-mucinous adenocarcinoma group (24.7%, 42/170) compared to the mucinous group (11.1%, 2/18). This discrepancy may be attributed to the higher rate of pathological vascular permeation invasion (VPI) in the non-mucinous adenocarcinoma cohort (21.0% vs 2.9%, *p* < 0.001). Clinically, a potential link between VPI and genetic mutation subtypes was observed; however, there is currently insufficient evidence to substantiate significant differences between VPI and mutation profiles.

This study has several limitations. First, it was based on data from a single center and employed a retrospective design. Second, while preoperative PET/CT imaging is a crucial and effective screening method for pure-solid-appearance lung cancer, only a small proportion of patients (6.9%, 40/579) in this study underwent PET/CT scanning prior to surgery. The relatively low rate of PET/CT utilization can likely be attributed to its high medical cost, which limits its widespread adoption as a routine diagnostic tool in China. Additionally, the evaluation of CT attenuation played a critical role in this study. However, the CT scans included in our retrospective analysis were conducted using varying technical acquisition parameters, and the CT attenuation protocols were not standardized across cases, leading to potential discrepancies in the results. To mitigate this variability, two radiologists independently measured CT attenuation for each case, with the average values used in subsequent analyses. Exploring this question through CT perfusion or DWI-IVIM sequence imaging in future studies would undoubtedly offer valuable insights. As we know, while invasive mucinous adenocarcinoma is thought to spread by tumor cells suspended in extracellular mucin, replacing air spaces—similar to the spread through air spaces (STAS) observed in other lung cancers—the concept of STAS was introduced in 2015 [2]. As the majority of data in our study was collected between 2015 and 2017, not all cases were assessed for STAS. Finally, the small number of recurrences in the mucinous adenocarcinoma subgroup limits the robustness of comparisons for site‑specific metastases, and the substantial difference in cohort size between the two groups may affect regression stability. These two aspects need to be noted.

In conclusion, two different pathological subtypes of lung adenocarcinoma have distinctive imaging features. Moreover, our findings demonstrate that survival outcomes and recurrence patterns differ significantly between mucinous and non-mucinous lung adenocarcinomas.

## Supplementary information

Supplementary information

## Data Availability

All data are available from the corresponding author upon request.

## Abbreviations

CEA: Carcinoembryonic antigen
CT: Computed tomography
EGFR: Epidermal growth factor receptor
IASLC/ATS/ERS: International Association for the Study of Lung Cancer/American Thoracic Society/European Respiratory Society
KRAS: Kirsten rat sarcoma viral oncogene
LMA: Lung mucinous adenocarcinoma
LVI: Lymphatic or vascular invasion
OS: Overall survival
PET: Positron emission tomography
RFS: Recurrence-free survival
TNM: Tumor–node–metastasis
VPI: Visceral pleural invasion
