# Supplementary material for: Radiological pure-solid appearance clinical stage I lung adenocarcinoma: a comparative study of mucinous and non-mucinous adenocarcinoma based on imaging features and survival outcomes
Source: Insights Imaging. 2026 Jul 23;17:192. doi: 10.1186/s13244-026-02353-x (PMC13396331; doi:10.1186/s13244-026-02353-x)
Supplement: Supplementary file 1 — Supplementary information [file 13244_2026_2353_MOESM1_ESM.pdf]

Radiological pure-solid appearance clinical stage I lung adenocarcinoma: a comparative study of mucinous and non-mucinous adenocarcinoma based on imaging features and survival outcomes

ELECTRONIC SUPPLEMENTARY MATERIAL

Supplement Table 1. Interobserver Agreement for CT Findings

			Agreement	
Characteristic	Radiologist 1	Radiologist 2	Coefficient	95% CI
Total tumor size (cm)	2.0±0.7	2.1±0.8	0.98[a]	0.97-0.99
Distributson (central)	15 (2.6)	16(2.8)	0.95[b]	0.91-0.98
Emphysema	116(20.0)	116(20.0)	1.00[b]	1.00-1.00
Air bronchogram	273(47.2)	270(46.6)	0.94[b]	0.90-0.97
CT value	32.8±17.0	33.7±17.9	0.92[a]	0.89-0.95

Note--Values are meant±SD or number with percentage in parentheses.
[a]Reported using intraclass correlation coefficient
[b]Reported using kappa coefficient

Supplement Table 2. Correlation between Recurrence Location and Mutation Site

Variable	Value
Brain metastasis	46
EGFR	32(69.6)
ALK	7(15.2)
KRAS	1(2.2)
Wild type	5(10.8)
Other	1(2.2)
Chest wall or pleura	44
EGFR	29(65.9)
ALK	4(9.1)
KRAS	2(4.5)
Wild type	8(18.2)
Other	1(2.3)
Contralateral lung	57
EGFR	35(61.4)
ALK	9(15.8)
KRAS	2(3.5)
Wild type	10(17.5)
Other	1(1.8)

Note. -Unless otherwise specified, data are numbers of recurrence patients, with percentages in parentheses.

EGFR = epidermal growth factor receptor, KRAS = kirsten rat sarcoma viral oncogene, ALK = anaplastic lymphoma kinase.

Supplement Table 3: Univariable Cox Proportional Hazard Regression Analysis of Factors Affecting Recurrence-Free Survival and Overall Survival

Variables	Recurrence-free survival		Overall survival	
	HR (95% CI)	*P* Value	HR (95% CI)	*P* Value
Gender, male [Female(ref.)]	1.3(0.9-1.7)	0.110	1.6(1.0-2.4)	0.042
Age, ＞62 [≤62(ref.)]	0.8 (0.6-1.1)	0.191	0.9 (0.6-1.4)	0.663
Smoking, Past or current [Never(ref.)]	1.0(0.7-1.5)	0.844	1.3(0.8-2.1)	0.303
CEA, ＞2.24 [≤2.24(ref.)]	1.6(1.2-2.1)	0.003	2.1(1.3-3.2)	0.001
Emphysema, [Absence(ref.)]	1.1 (0.8-1.6)	0.564	1.6(1.0-2.5)	0.063
Distribution, [Central(ref.)]	0.3(0.2-0.5)	0.000	0.5 (0.2-1.3)	0.136
Location, [Right upper lobe(ref.)]		0.178		0.048
Right middle lobe	1.0 (0.6-1.7)	0.903	0.7 (0.3-1.6)	0.446
Right lower lobe	0.6 (0.4-1.0)	0.029	0.3 (0.1-0.7)	0.003
Left upper lobe	0.9(0.6-1.3)	0.521	1.0(0.5-1.7)	0.870
Left lower lobe	0.8(0.5-1.1)	0.182	0.9(0.5-1.6)	0.787
CT value, ＞34 [≤34(ref.)]	1.8(1.3-2.4)	0.000	1.3(0.9-2.1)	0.174
Clinical T stage, [T2a(ref.)]		0.002		0.335
T1a	0.1 (0.03-0.5)	0.003	0.0 (0.0-∞)	0.959
T1b	0.9(0.5-1.4)	0.498	0.8(0.4-1.5)	0.412
T1c	1.2 (0.8-1.9)	0.404	1.7(0.6-2.2)	0.636
Air bronchogram, [Absence(ref.)]	0.5(0.3-0.6)	0.000	0.4(0.3-0.7)	0.000
Histology, [Mucinous(ref.)]	3.6(2.2-5.8)	0.000	4.0(1.8-8.6)	0.000
Pathological nodal involvement, [Negative (ref.)]	3.7(2.7-5.0)	0.000	3.2(2.1-5.0)	0.000
Lymphatic/Vascular involvement, [Negative (ref.)]	2.6(1.4-4.7)	0.003	3.0(1.4-6.5)	0.006
Gene mutation, [No (ref.)]	1.0(0.7-1.4)	0.825	0. 8(0.5-1.3)	0.346
Genetic Information, Mutated [Wild-type (ref.)]		0.056		0.854
EGFR	1.2 (0.8-1.7)	0.303	0.8 (0.5-1.4)	0.461
KRAS	0.5 (0.3-0.9)	0.028	0.9 (0.4-1.8)	0.768
ALK	1.1 (0.7-1.9)	0.714	0.6 (0.3-1.5)	0.279
other	0.8(0.2-3.2)	0.717	0.0 (0.000-∞)	0.963
Operative procedure, non-lob [Lob (ref.)]	0.8(0.5-1.3)	0.324	1.1 (0.6-2.0)	0.740
Postoperative treatment, [No (ref.)]	2.2(1.6-3.0)	0.000	1.2 (0.8-1.9)	0.322

Note. -HR=hazard ratio, CI =confidence interval, CEA = carcinoembryonic antigen, EGFR = epidermal growth factor receptor, KRAS = kirsten rat sarcoma viral oncogene, ALK = anaplastic lymphoma kinase.
